# Why is temperature sensitivity important for the success of common respiratory viruses?

**DOI:** 10.1002/rmv.2153

**Published:** 2020-08-10

**Authors:** Ronald Eccles

**Affiliations:** ^1^ Emeritus Professor, Cardiff School of Biosciences Cardiff University UK

## Abstract

This review explores the idea that temperature sensitivity is an important factor in determining the success of respiratory viruses as human parasites. The review discusses several questions. What is viral temperature sensitivity? At what range of temperatures are common respiratory viruses sensitive? What is the mechanism for their temperature sensitivity? What is the range of temperature along the human airway? What is it that makes respiratory viruses such successful parasites of the human airway? What is the role of temperature sensitivity in respiratory zoonoses? A definition of temperature sensitivity is proposed, as “the property of a virus to replicate poorly or not at all, at the normal body temperature of the host (restrictive temperature), but to replicate well at the lower temperatures found in the upper airway of the host (permissive temperature).” Temperature sensitivity may influence the success of a respiratory virus in several ways. Firstly; by restricting the infection to the upper airways and reducing the chance of systemic infection that may reduce host mobility and increase mortality, and thus limit the spread of the virus. Secondly; by causing a mild upper airway illness with a limited immune response compared to systemic infection, which means that persistent herd immunity does not develop to the same extent as with systemic infections, and re‐infection may occur later. Thirdly; infection of the upper airway triggers local reflex rhinorrhea, coughing and sneezing which aid the exit of the virus from the host and the spread of infection in the community.

## 1 INTRODUCTION

“Respiratory tract infections are the most common infections to afflict mankind and are responsible for an enormous burden of disease, ranging from trivial mild common colds, to severe fatal pneumonias.”^1^ This statement begs the question, why are respiratory infections so common and respiratory viruses such successful parasites?

There are several reasons why viruses commonly infect the airway and one of the first is that the airway is easily accessible to viruses. An adult breathes in 10 000‐15 000 L of air a day. A 2‐year‐old child has a respiratory rate at rest of 26 breaths per minute which equates to 37 000 breaths each day,^2^ thus all the respiratory tract is continuously exposed to potential infection from large volumes of inspired air. However, the most common respiratory infections are restricted to the upper airways, and “All known respiratory viruses are able to produce the illness complex recognized as the common cold.”^1^ The restriction of the most common respiratory infections to the upper airway is related to the “temperature sensitivity” of respiratory viruses and this review will put forward the idea that “temperature sensitivity” is an important factor for the success of respiratory viruses as parasites of the airway. This idea has not been fully explored before in the literature, although a medical hypothesis has discussed that the seasonality of respiratory viruses may be due to their temperature sensitivity.^3^ Most of the interest in the temperature sensitivity of respiratory viruses has focused on the development of vaccines and there is little discussion on how temperature sensitivity influences the success of the virus as a parasite of the airway.^4^, ^5^, ^6^, ^7^ Recent reviews on respiratory viruses do not mention airway temperature sensitivity.^8^, ^9^, ^10^

This review will explore the idea that temperature sensitivity is an important factor in determining the success of respiratory viruses as human parasites by addressing a series of related questions.

## 2 SEARCH STRATEGY

References for this review were identified through searches on PubMed using the terms “temperature sensitivity” or “temperature sensitive” linked with “virus,” “influenza,” “rhinovirus,” “respiratory virus,” and domestic chicken, domestic pig, pangolin, civet cat, and dromedary camel linked with body temperature. Google Scholar was also used to search for references using the same search terms. The bibliographies of articles were searched for relevant references and the Web of Science was used to search for citations to references.

## 3 WHAT IS VIRAL TEMPERATURE SENSITIVITY?

Viruses may be inactivated at high temperatures and this is a common form of sterilization but this is not what is normally meant by viral temperature sensitivity.^11^ Similarly, the rate of inactivation of viruses in the environment on surfaces and droplets may be related to ambient temperature,^12^ but this also is not what is meant by “temperature sensitive.” All living systems are sensitive to changes in temperature, as temperature influences the rate at which biochemical reactions proceed, but viruses can only be considered as biochemically active when they are replicating inside a host cell, and it is during this phase of infection that viruses are temperature sensitive.

When human viruses are studied in cell and tissue culture there is a range of temperature over which they replicate and this temperature sensitivity was nicely defined by Richman and Murphy in 1979. “Viruses that replicate well at low (permissive) temperatures and poorly at higher (restrictive) temperatures are defined as temperature sensitive (ts).”^13^ What is implied in this definition of virus temperature sensitivity is that the higher temperature is the normal body temperature of the host and that viruses that replicate poorly at body temperature are less virulent than those that can replicate freely at body temperature. This understanding of temperature sensitivity was first put forward by Andre Lwoff in 1959 who proposed that the virulence of a microorganism was dependent on the temperature sensitivity of their replication and thus on the body temperature of their host.^14^

The temperature sensitivity of viruses was first discovered for plant viruses in 1921 when it was observed that tobacco plants infected with what was later identified as tobacco mosaic virus, developed typical signs of disease at temperatures between 20°C and 30°C but little signs of disease at temperatures above 30°C and none at 37^o^C.^15^, ^16^ Temperature sensitive animal viruses were later described and soon a wide range of human viruses were shown to be temperature sensitive when cultured, such as polio viruses, adenoviruses, influenza viruses, measles, rabies, and there was growing interest in utilizing the temperature sensitivity of human viruses to develop live attenuated virus vaccines.^13^

Looking through the literature there is much use of the term “temperature sensitivity” but this term is not often defined, and the best definition in the literature is the one given above by Richman and Murphy in 1979.^13^ With further understanding of the properties of viruses since 1979 a better definition of temperature sensitivity may be used in this review on respiratory viruses:

*Virus “temperature sensitivity” is the property of a virus to replicate poorly or not at all at the normal body temperature of the host (restrictive temperature) but to replicate well at the lower temperatures found in the upper airway of the host (permissive temperature)*.

The temperature sensitivity of a virus is often discussed as a means of attenuating the virulence of a virus by looking for temperature sensitive phenotypes amongst wild strains of a virus, or using serial cultures of the virus at lower and lower temperatures in order to develop a live attenuated vaccine,^5^, ^13^, ^17^ but there is little discussion in this literature about how wild viruses may benefit as parasites by evolving a phenotype for temperature sensitivity.

## 4 AT WHAT RANGE OF TEMPERATURES ARE COMMON RESPIRATORY VIRUSES SENSITIVE?

Right from the discovery of respiratory viruses, temperature has been known to have a major influence on the replication of respiratory viruses. The first cell cultures for an unknown virus which was later to be described as a rhinovirus were made unsuccessfully at 36°C and it was only when the temperature was reduced to 32°C that the viruses replicated well, and the investigators speculated that the cooler conditions for culture were like those at the surface of the nasal mucosa.^18^ The first cultures of a novel virus later to be described as a coronavirus were also made at the cooler temperature of 33°C rather than closer to human body temperature of 37^o^C.^19^

The common respiratory viruses include adenovirus, enterovirus, human coronavirus, human metapneumovirus, rhinovirus (RV), influenza, parainfluenza, and respiratory syncytial virus (RSV).^20^ All these common respiratory viruses replicate best at a temperature close to that of the human upper airway which is between 32°C and 34^o^C.^21^ The term “respiratory viruses” is not an exact term as viruses are opportunist and may infect any tissue and organ in the host that allows virus entry. In this review, the term “respiratory viruses,” will mean viruses that predominantly enter and exit the host via the airway.

Table 1 illustrates the temperatures at which a range of common respiratory viruses were cultured. One of the first demonstrations of temperature sensitivity in a respiratory virus was made by Tyrrell and Parsons in 1960 when attempting to culture a virus responsible for causing a common cold syndrome of disease, and this virus was later named as a rhinovirus.^18^, ^22^, ^23^ The early experiments at culturing rhinoviruses discovered that the virus replicated better at 33°C than 37°C but as more and more rhinoviruses were discovered it was found that some of the 100 or so serotypes replicated well at 37°C and this may explain why some rhinovirus infect the lower airways and cause exacerbations of asthma.^1^, ^24^, ^25^

**TABLE 1:** Temperatures at which a range of common respiratory viruses were cultured

Virus	Culture temperature	Reference
Adenovirus	32‐35°C	30
Human Coronavirus	32‐33°C	26 19, 27
Rhinovirus	32‐33°C	24 18
Human Influenza A	33‐37°C	34
Avian Influenza	40°C	34
Human Metapneumovirus	33°C	31
Parainfluenza	34°C	29
Respiratory Syncytial Virus	32–33°C	28
Enterovirus (respiratory illness)	33°C	32 33

Since the discovery of the temperature sensitivity of rhinoviruses other respiratory viruses have also been found to be temperature sensitive and this temperature sensitivity is a characteristic of the most common respiratory viruses such as corona viruses,^19^, ^26^, ^27^ respiratory syncytial viruses,^28^ parainfluenza viruses,^29^ adenoviruses,^30^ human metapneumovirus^31^ and some enteroviruses that infect the respiratory tract.^32^, ^33^

The common respiratory viruses replicate best at the cooler temperatures of the human upper airways between 32°C and 35°C and viruses such as avian influenza viruses are not successful in spreading from domestic birds to humans despite the many close contacts and occasional infections, as they are adapted to replicating in the avian gut at the normal avian temperature of 40^o^C.^34^

It has been known for some time that human influenza viruses vary in their temperature sensitivity with those adapted to the cooler human airway causing mild disease and those adapted to higher temperatures causing more serious lower respiratory tract infections.^13^, ^35^That is why in Table 1 the culture temperature for human influenza ranges from 33°C to 37°C.

In summary the common respiratory viruses have a permissive temperature similar to the temperature of the human airway 32°C‐35°C as illustrated in Table 1.

## 5 WHAT IS THE MECHANISM OF TEMPERATURE SENSITIVITY?

There is no one mechanism of temperature sensitivity, as a wide range of viruses from plant to human types exhibit temperature sensitivity and for each virus most of the proteins it produces in the host cell will exhibit some degree of temperature sensitivity. This review focuses on the temperature sensitivity of common respiratory viruses, and the mechanism of temperature sensitivity in one of the most studied viruses, influenza A will be discussed below as an example of how this sensitivity to temperature is determined.

The temperature sensitivity of respiratory viruses first became of interest in attempts to develop live attenuated vaccines for influenza A and RSV as the most serious causes of human respiratory illness. The influenza A virus was an obvious target for developing a vaccine as temperature sensitive strains of virus could be found as wild types or could be developed by serial culture at low temperatures. The influenza A virus also had a great advantage in having a segmented genome so that temperature sensitive genes could be added to the genome without disrupting the genes for important functions such as viral attachment. In those early days of developing a live attenuated vaccine for influenza A there was little if any understanding of the mechanism that regulated temperature sensitivity and most studies discussed “genetic lesions” as being responsible for temperature sensitivity.^36^, ^37^

By 1979 those involved in developing vaccines from temperature sensitive viruses were speculating that the “genetic mutation” specified a protein with a tertiary structure that was relatively thermolabile compared with that of the wild type protein and that the thermolabile protein did not function normally at the higher temperatures.^13^

In 1980 two genes responsible for production of influenza viral polymerase proteins P1 and P3 were reported to be involved in determining the temperature sensitivity of wild type influenza viruses.^38^

After searching for a specific mechanism to explain the temperature sensitivity of influenza A viruses, researchers realized that all the proteins produced by the virus could impart some degree temperature sensitivity and this conclusion for viruses in general had been reached years before in 1959 by Richman and Murphy who stated that “attenuation can result from a temperature sensitive mutation in any gene.”^13^ The impact of temperature sensitivity on the replication of the virus was dependent on the importance of the viral protein in the replication of the virus and escape of the virus from the host cell.^17^

The RNA‐dependent RNA polymerase enzyme of influenza A, that is responsible for transcribing and replicating the negative sense segmented viral genome has been put forward as a key determinant of viral pathogenicity and host range for the influenza A virus.^39^ Although the polymerase enzyme appears to be an important target for developing temperature sensitive influenza A strains, a recent review states that other viral proteins can also be considered as targets when developing live attenuated influenza A vaccines.^17^

The mechanism of temperature sensitivity in respiratory viruses can therefore be related to the structure of viral enzymes and even slight changes in the structure such as substitution of single amino acids can influence the temperature sensitivity of the enzyme.^40^ Exactly how the small changes in enzyme structure influence the temperature sensitivity of the enzyme is at present only speculation but it seems the best explanation is that the substitution of a single amino acid causes a change in the folding of the enzyme that influences the activity of the enzyme.^40^

In summary, the mechanism of temperature sensitivity is not known but may be related to small changes in the structure of key viral enzymes that influence the folding of the enzyme and its tertiary structure.

## 6 WHAT IS THE RANGE OF TEMPERATURE ALONG THE HUMAN AIRWAY?

Ambient air over the wide range of climates inhabited by man is warmed by the nose and upper airway to a temperature of 37°C at the level of the alveoli.^41^ The nose and upper airway have a great capacity to warm the inspired air and even at an inspired air temperature of −17°C the air temperature is 34°C at the level of the bronchi and reaches 37°C before the alveoli.^42^

**FIGURE 1:**
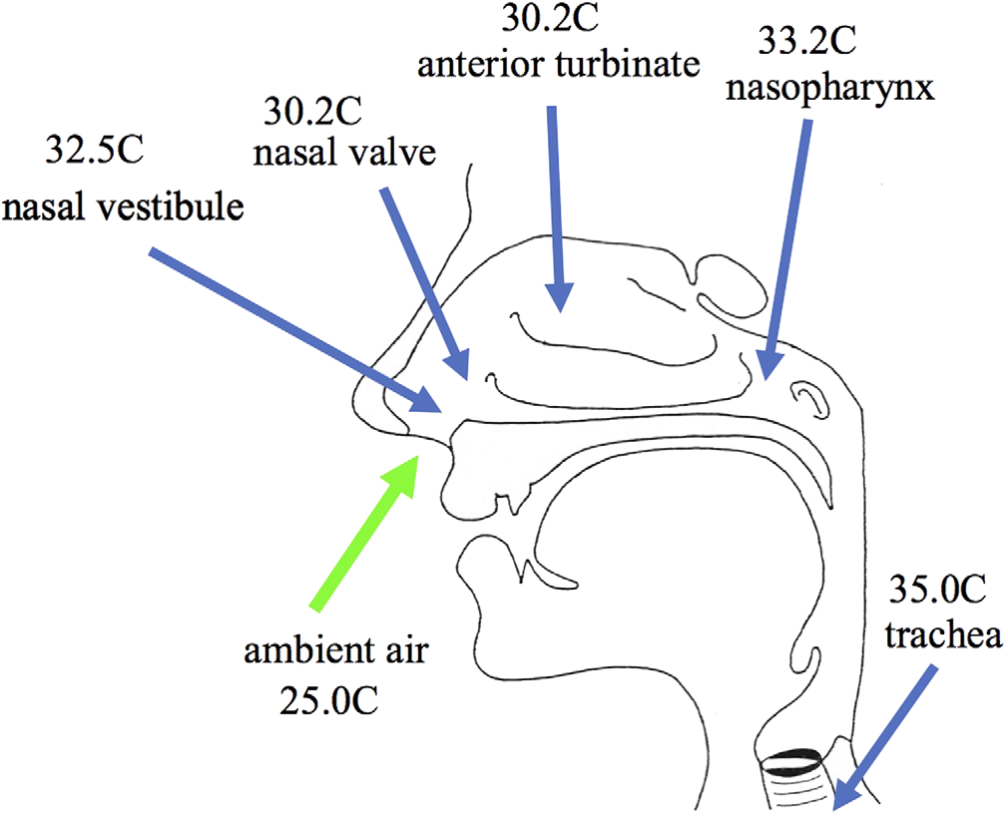
Airway temperatures. Nasal temperatures are nasal mucosal temperatures measured during inspiration of ambient air at 25C (Lindemann et al 2002). Tracheal temperature is for inspiratory air temperature (Cole 1988) See text for details

The temperature of the mucosa of the airway depends on the temperature of the inspired air, the site in the airway where the measurement is made and whether the measurement is made during inspiration or expiration. Mean nasal mucosal temperature ranged from 30.2°C to 34.4°C in a study on 15 healthy subjects breathing ambient air at 25°C.^43^ Figure 1 illustrates the range of temperature along the human airway from the entrance of the nose at the nasal vestibule(32.5°C) to the trachea(35.0°C). The temperatures in Figure 1 from the nasal vestibule to the nasopharynx are for mucosal temperature during inspiration of air at 25°C as studied in 15 healthy subjects,^43^ and the tracheal temperature is the mean inspired air temperature from 17 different studies as reported by Cole 1988.^41^ A study by Keck et al (2000) on 50 healthy subjects breathing air at 25°C reported a range of nasal mucosal temperatures similar to those shown in Figure 1 (nasal vestibule 25.3°C, nasal valve 29.8°C, middle turbinate 32.3°C, nasopharynx 33.9°C) and variations between the studies may be due to differences in the thermocouples used in the two studies and differences in their positioning.^21^

In summary the range of mucosal temperature along the human airway when breathing air at 25°C is from around 30°C‐34°C in the nose to 37°C in the lungs.

## 7 WHAT IS IT THAT MAKES RESPIRATORY VIRUSES SUCH SUCCESSFUL PARASITES OF THE HUMAN AIRWAY?

The common cold syndrome of disease caused by respiratory viruses is the most common human disease; a mild disease, that is more of a nuisance to the host than a serious illness.^44^, ^45^ The common cold syndrome is a mild disease because the respiratory viruses have a permissive temperature sensitivity close to that found in the human upper airways 30°C‐34°C and a restrictive temperature sensitivity that confines most of their replication to the upper airways and inhibits replication at 37°C therby reducing the incidence of serious systemic and lower airway infection.

Respiratory viruses that are temperature sensitive cause a mild illness that allows the host to continue to function, more or less normally, and go to school and work and interact socially with other potential hosts, and therefore effectively spread the infection. Serious systemic or lower respiratory infections would trigger a greater immune response with more severe cytokine mediated responses such as fever, malaise, tiredness, and what is generally termed “sickness behavior,” and this would restrict the activity of the host and increase mortality, which would limit spread of the virus.^46^, ^47^, ^48^

Common symptoms of the common cold are runny nose, sneezing and cough. The mechanisms of these symptoms have been previously reviewed.^44^ The symptoms are triggered by the host defensive response to the viral infection with the generation of inflammatory mediators such as bradykinin and prostaglandins that stimulate sensory nerves in the upper airway to cause reflex nasal secretions and sneezing by stimulating trigeminal nerve endings in the nose, and cough by stimulating vagal nerve endings in the larynx and trachea. The host response to upper airway viral infection provides the exit mechanism for respiratory viruses as they are transmitted in respiratory fluid on fomites that can contaminate hands, and in airway fluid expelled as aerosols by coughs and sneezes.^49^, ^50^ Asymptomatic respiratory infections are common but these infections are less likely to spread from the host as there will be no spread of respiratory fluid from coughing and sneezing.^51^, ^52^

Restricting infection to the upper airways may not only increase the spread of infection by coughing and sneezing but may also benefit the virus by locating infection in a cooler part of the airway that has a less efficient antiviral defence when compared to the warmer lower airways. Studies on human and mouse airway epithelial cells show that the interferon response to infection is inhibited at the lower temperatures found in the upper airways compared to warmer temperatures found in the lower airways.^53^, ^54^

## 8 WHAT IS THE ROLE OF TEMPERATURE SENSITIVITY IN RESPIRATORY ZOONOSES?

Zoonoses are diseases that can be transmitted from animals to humans, such as the recent well researched outbreaks of human respiratory infections such as avian influenza (H5N1),^55^ severe acute respiratory syndrome (SARS‐CoV),^56^ Middle East respiratory Syndrome (MERS‐CoV),^57^ and COVID‐19 (SARS‐CoV2).^58^

The acquisition of temperature sensitivity is key to the success of a respiratory virus and this is well illustrated with emerging avian influenza H5N1. Influenza viruses originating from avian sources initially do not succeed as human parasites because they have high virulence and they lack the genotypes that confer temperature sensitivity, but as they acquire temperature sensitivity they become more successful human parasites.^59^ Zoonotic transmission of avian influenza also involves a change in the binding specificity of hemagglutinin from Neu5Acα2‐3Gal linked (α2‐3) to Neu5Acα2‐6Gal linked (α2‐6) glycans which is essential for the crossover of the viruses from avian to human hosts.^60^

It is not the remit of this review to specifically discuss SARS‐CoV‐2 coronavirus but recent research indicates that this virus does have some temperature sensitivity that may influence its transmission according to environmental temperature^61^ but at present there is little understanding of how temperature sensitivity influences its infection of the airway. Tissue cultures of SARS‐CoV‐2 have been made at 37^o^C^62^ whereas the four coronaviruses that cause the common cold syndrome are incubated in cultures at 33°C.^63^ The temperature sensitivity of SARS‐CoV‐2 is important for its role as human parasite, because a permissive temperature close to that of the human upper airway such as 33°C would allow easy exit from the airway in respiratory fluid, whereas a permissive temperature of 37°C would tend to restrict infections to the lower airway and cause more serious illness that could restrict transmission. The exact origins of SARS‐CoV‐2 are not yet clear but it is closely related to bat coronaviruses and may have caused human infection via an intermediate host, the Chinese pangolin.^64^ Bats and pangolins are mammals, but they have body temperatures different from man, 15°C‐41°C in bats, depending on level of activity, and around 33°C in pangolins,^65^ and it is not understood how the animal origin for SARS‐CoV‐2 influences its temperature sensitivity.

Table 2 lists some respiratory virus zoonoses with their animal of origin and the body temperature of each animal host. It is interesting that animals with a high body temperature (41.8°C) such as the domestic chicken which hosts influenza H5N1 do not transmit well to human hosts, whereas SARS‐CoV‐2 which does transmit well, may have the Pangolin as a host^66^, ^67^with a body temperature of 33.4°C which is similar to that of the human upper airway.

**TABLE 2:** Some respiratory virus zoonoses, with their animal of origin and the body temperature of each animal host

Animal	Zoonotic virus	Body temperature	Reference
Domestic Chicken	Influenza H5N1	41.8C	^69^
Domestic Pig	Influenza H1N1	39.2C	^70^
Dromedary Camel	SARS‐CoV	37.5C	^71^
Palm Civet	MERS‐CoV	36.5	^72^
Chinese Pangolin	SARS‐CoV‐2	33.4C	^73^

Temperature sensitivity is only one factor to be considered when discussing the possible success of zoonoses, as this is a complicated area of virology, and what is not often clear is the route of transmission from animal to man, and if this is via body fluids such as blood or faeces, or via respiratory droplets entering the human airway.

## 9 DISCUSSION

**FIGURE 2:**
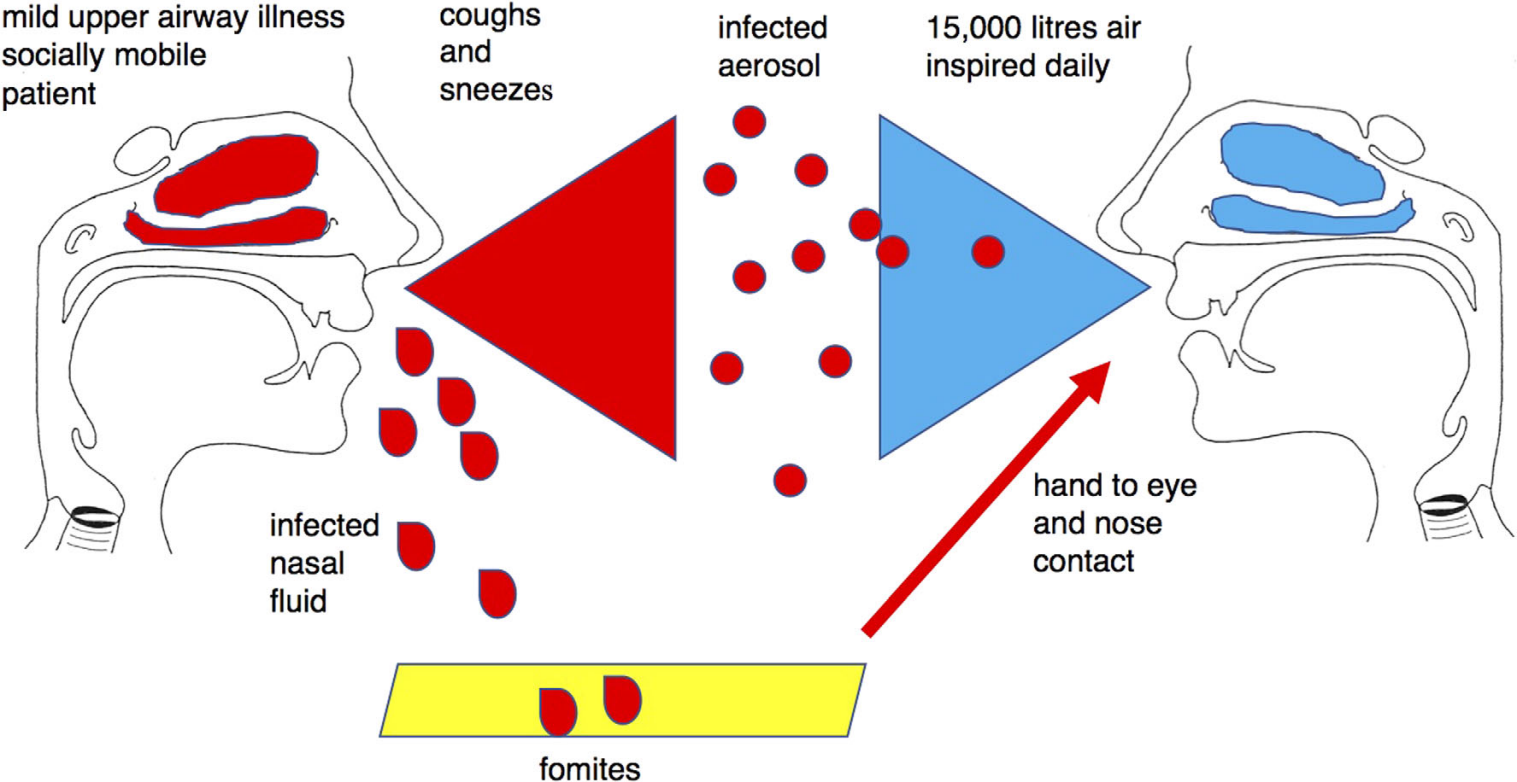
Diagram to illustrate transmission of respiratory viruses. The Infected host is illustrated on the left in red and the susceptible host on the right in blue. Infectious nasal fluid can reach the susceptible host by aerosols generated by coughs, or via fomites and hand transmission the nose and eye

The human airways are an obvious target for viruses because of the ease of access, but once in the airway and replicating the viruses must have a means of exit to reach another host, as the airway defences will eventually overwhelm the virus or the host will eventually die if the infection is not controlled. Figure 2 illustrates how respiratory viruses are transmitted in expelled respiratory fluid by coughs and sneezes or on fomites. The important factor in the transmissibility of the virus is that the virus is mainly restricted to infecting the upper airway because of temperature sensitivity.

All the common respiratory viruses such as rhinoviruses exhibit temperature sensitivity and emerging respiratory viruses such as influenza H5N1 which are not temperature sensitive in a human host have a high virulence and do not spread readily amongst humans.^59^ The success of respiratory viruses is not limited to temperature sensitivity as other factors are also important such as the ability of RNA viruses to rapidly evolve many different genotypes.^68^ Rapid evolution of respiratory viruses not only enables the virus to regularly infect the same host but also allows the virus to rapidly acquire characteristics such as temperature sensitivity.

The definition of “success” used in this review is that the virus is common amongst its host population and that it persists in the population. Temperature sensitivity influences the success of a respiratory virus in several ways. First; temperature sensitivity limits serious illness of the host by restricting the infection to the upper airways and reduces the chance of lower airway and systemic infections that may reduce host mobility and increase mortality, and thus limit the spread of the virus. Second; a mild illness of an upper airway infection causes a limited immune response compared to systemic infection, which means that persistent herd immunity does not develop to the same extent with upper airway infections compared to systemic infections and re‐infection may occur later. Third; infection of the upper airway triggers local reflex rhinorrhea, coughing, and sneezing which aid the exit of the virus from the host and the spread of infection in the community.

An important factor in the exit of the virus from the upper airway is the triggering of rhinorrhea by the viral infection. Nasal secretions in health are normally cleared from the nose by mucociliary clearance and swallowed, but viral infection of the upper airways causes an increase in nasal secretions and formation of plasma exudate^44^ and this infected watery fluid drips from the nostril to contaminate hands and surfaces and can also be expelled by coughs and sneezes.

## 10 CONCLUSION

The temperature sensitivity of respiratory viruses should be considered as an important factor in determining their success as parasites of the human airway.

## CONFLICT OF INTEREST

The author has no conflicts of interest to declare regarding this review article.

## Data Availability

Data sharing not applicable to this article as no datasets were generated or analysed during the current study
